# Progress in Diagnosis and Treatment of Neonatal Sepsis: A Review Article

**DOI:** 10.31729/jnma.7324

**Published:** 2022-03-31

**Authors:** Pratibha Yadav, Shailendra Kumar Yadav

**Affiliations:** 1 Department of Pediatrics, Second Affiliated Hospital of Kunming Medical University, Kunming, Yunnan 650101, People's Republic of China; 2 Trishuli Hospital, Bidur, Nuwakot, Nepal

**Keywords:** *cytokines*, *immunoglobulin*, *neonatal sepsis*, *procalcitonin*

## Abstract

Neonatal sepsis is a serious condition in which the pathogens infiltrate the bloodstream, multiply and produce toxins causing deleterious effects to the health of neonates. It is divided into two types on the basis of the time of onset. Early onset sepsis occurs within 72 hours of birth and late onset sepsis begins after 72 hours of delivery. Neonatal sepsis continues to be a common and significant health care burden, especially in very low birth weight infants (with birth weight less than 1500 grams). Though intrapartum antibiotic prophylaxis has decreased the incidence of early-onset group B streptococcal infection dramatically, it still remains a major cause of neonatal sepsis. As the signs and symptoms of neonatal sepsis are nonspecific, early diagnosis and prompt treatment remain a challenge.

## INTRODUCTION

Sepsis is a systemic infection caused by pathogens invading the blood circulation, growing, and reproducing in it, and producing toxins leading to serious health conditions and neonatal death. The incidence rate is about 0.1-1% of live birth infants, which accounts for 16.4% of very low birth weight (VLBW) infants. The incidence of long-term hospitalisation is as high as 30% and the mortality is 10-50%. Neonatal sepsis was divided into early-onset sepsis (EOS) and late-onset sepsis (LOS). EOS refers to the onset time within 72 hours after birth, often associated with prenatal and intrapartum infection. The onset time of LOS is 72 hours after birth, usually hospital infection or community-acquired infection.^1,2^

## RISK FACTORS OF NEONATAL SEPSIS

### 1. EARLY ONSET SEPSIS

In this condition, the mother is infected before delivery. The pathogen can infect the foetus through the placenta. It can also cause aspiration pneumonia by inhaling bacteria-contaminated amniotic fluid or vaginal secretion in the uterus or during delivery after premature rupture of membranes. Martus JA, et al. showed that the main risk factors for EOS were maternal history of abortion, WBC >15 x 10^9^/L, premature rupture of membranes >18 hours, C-reactive protein (CRP) >1.5 mg/l, body temperature >38°C, prenatal use of antibiotics, chorioamnionitis, endometritis, neonatal low Apgar score and low gestational age (the incidence of full-term infants was 0.6% and the incidence of premature infants <28 weeks was 16.6%). In addition, intracranial haemorrhage is also closely related to sepsis.^3,4^

The study on risk factors of EOS in LBW infants conducted by Israel neonatal collaboration network found that lack of prenatal care, absence of amnionitis in care of premature rupture of membranes >24 hours or amnionitis care of premature rupture of membranes <24 hours, and resuscitation in the delivery room were all associated with EOS, but not with the use of corticosteroids and gestational age. If premature rupture of membranes and amnionitis exist at the same time the risk is eight times higher than that when they exist alone.^5,6^

### 2. LATE ONSET SEPSIS

It is common in neonates with central venous catheterization in the neonatal intensive care unit (NICU), especially in those with extremely low birth weight (ELBW) <1000 g premature infants and those with total parenteral nutrition and mechanical ventilation. In NICU, because of the long hospital stay, and critically ill newborns, although countries have taken a series of preventive measures.^7,8^ Nosocomial infection is still a problem in NICUs all over the world. The incidence of neonatal nosocomial infection is 30% and 40% of neonatal deaths occur in developing countries,^9^ Although nosocomial infection is difficult to eliminate, it can be prevented and controlled. Brazilian scholars have studied the risk factors of nosocomial infection in the NICU. In addition to placenta transmission, they classified all intrapartum and post-hospital infections as nosocomial infections. The nosocomial infections rate was 34% of which 68.2% was bloodstream infection. Maternal venereal disease, placental abruption, BW <1500 g, parental nutrition, subcutaneous indwelling catheter, central venous catheterization, and mechanical ventilation were independent risk factors.^10,11^

## PATHOGENIC BACTERIA OF NEONATAL SEPSIS

There are different reports about the pathogens of neonatal sepsis at home and abroad. Group B *Streptococcus* (GBS) was the main pathogen of EOS reported in developed countries in the 1970s, and the mortality was 55%.^12J3^ Since 1992, the American Academy of Paediatrics has formulated guidelines for the use of antibiotics during labour to prevent GBS infection in newborns. In 2002, the guidelines recommended screening for GBS infection in pregnant women at 3537 weeks of gestation and recommended penicillin as the first-line drug for intrapartum antibiotics.^14,15^ After that, EOS caused by GBS infection was greatly reduced. According to US statistics, the incidence rate dropped from 2/1000 live births in 1996 to 0.36/1000 in 2005. Other Gram-positive bacteria causing EOS were *Staphylococcus aureus, Enterococcus, Streptococcus pneumonia,* etc. In VLBW, 65% of mothers had used antibiotics before delivery, so GBS infection decreased gradually. No matter EOS or LOS, *Escherichia coli* infection showed an upward trend, especially in children with fever symptoms. Other gram-negative bacteria causing neonatal sepsis include *Klebsiella pneumoniae*, *Enterobacter, Salmonella,* etc.^16-18^

In recent years, the surveillance of septic pathogens in many hospitals in China showed that *Staphylococcus* was the main pathogen, followed by *Escherichia coli* and GBS was rare. With the widespread use of antibiotics, the incidence of neonatal sepsis caused by opportunistic pathogens increased gradually in recent years and the drug-resistant strains increased significantly, showing a trend of multi-drug resistance. Coagulase-negative *Staphylococcus* (CNS) is mainly found in premature infants, especially in patients with long term arteriovenous catheterization. *Staphylococcus aureus* is mainly found in skin suppurative infection, and gramnegative bacilli, mainly *Escherichia coli*, are mainly common in prenatal or intrapartum infection. Gramnegative bacilli such as *Pseudomonas aeruginosa, Klebsiella pneumoniae* and *Serratia* were the main pathogens in children with tracheal intubation and mechanical ventilation. With the variety of antibiotics used, pathogenic bacteria have changed a lot in recent ten years.^19,20^

The infants who suffered from bacterial infections do not show the specific signs and symptoms of viral illness of either adenovirus or enterovirus. The symptoms of viral illness are very fast within a few days after birth.^21,22^

## CLINICAL SYMPTOMS

The clinical symptoms of neonatal septicemia are often non-specific, and blood culture is the gold standard for the diagnosis of septicemia, but blood culture cannot produce immediate results. Some children have used antibiotics before blood culture, which brings some difficulties to early diagnosis. WBC count, neutrophil classification and CRP elevation are not specific to septicemia, so scholars at home and abroad are committed to exploring more sensitive indicators for early diagnosis. Clinical manifestations range from subtle symptoms to profound septic shock. Signs and symptoms of sepsis are nonspecific and include temperature instability, mostly fever, irritability, lethargy, tachypnea, grunting, hypoxia, poor feeding, tachycardia, poor perfusion and hypotension.^23^

## INDICATORS

### 1. PROCALCITONIN (PCT)

PCT is a procalcitonin precursor, a glycoprotein containing 116 amino acid residues, which is produced in the liver and cannot be detected in normal human plasma. Its production is similar to that of acute-phase protein production, and the increase is related to bacterial infection.^24^ The plasma PCT of children with systemic bacterial infection/septicemia is increased, while the PCT of children with viral infection or bacterial colonisation is normal or slightly increased. Its specificity, sensitivity and predictive value were better than those of CRP, IL-6 and WBC count. The PCT of newborns increased physiologically on the first day after birth, and the peak value returned to normal at 18-30 hours after birth and returned to normal at 48 hours after birth, and was similar to that of adults 3 days later. In animal experiments, it was found that the peak value of PCT, 12 hours, could be detected in plasma at 2 hours after injection of endotoxin, which was more than 100 times the normal value. However, CRP increased at 12 hours after infection and peaked at 20-72 hours. After infection control, PCT and CRP decreased to normal after 2 to 3 days and 3 to 7 days, respectively. PCT is often significantly increased in children with septic shock, which is related to the severity of organ failure and mortality. The decrease of PCT level 24 hours after treatment indicates a good prognosis.^25^ Although many studies at home and abroad support that the increase of plasma PCT in systemic bacterial infection/septicemia is earlier than the changes of body temperature, WBC count and CRP. It can be used as an important index for early diagnosis and evaluation of curative effect in patients with severe systemic infection or failure, but because PCT fluctuates widely within 3 days after birth, the diagnostic value of EOS is still controversial.^26,27^

### 2. C-REACTIVE PROTEIN (CRP)

CRP levels are raised in inflammatory conditions including sepsis. There are so many noninfectious inflammatory conditions in which CRP is elevated, including maternal fever, foetal distress, stressful delivery, perinatal asphyxia, meconium aspiration and intraventricular haemorrhage. CRP itself cannot diagnose neonatal sepsis because of low sensitivity and specificity. If CRP remains normal persistently then bacterial neonatal sepsis is unlikely. It also helps in monitoring the duration of antibiotic therapy in sepsis.^28^

### 3. CYTOKINES

Cytokines such as IL and tumour necrosis factor (TNF) are important inflammatory mediators of systemic infection. IL shock is the main factor that induces B cells to secrete immunoglobulin and T cells to activate and proliferate, and it is also the main cytokine that stimulates hepatocytes to synthesise and release acute-phase proteins. It was released 60 minutes after infection, and the symptoms of septicemia increased significantly 2 days before the occurrence of septicemia, which can be used as an index for early diagnosis of septicemia. IL-8 is mainly produced by endothelial cells, mononuclear macrophages and T cells, and has chemotaxis to neutrophils and lymphocytes. TNF is produced by macrophages and activated cells. It has the effect of anti-infection, causing inflammation and anti-tumor.29 Turkish scholars compared the significance of PCT, CRP, IL-6, IL-8 and TNF-Q in the diagnosis and prognosis of neonatal septicemia.^30^ The results showed that the above markers in the septicemia group were significantly higher than those in normal newborns before treatment. The authors believe that PCT and TNF are higher than CRP, IL-6 and IL-8 in sensitivity, specificity, positive predictive value, negative predictive value and diagnostic value in the diagnosis of neonatal septicemia.^31^

### 4. INHIBITORY PROTEIN

Inhibitory protein is structurally related to the serine protease inhibitor family, which is mainly synthesised in the liver and plays an important role in antiinflammation and infection. The plasma level in adult septicemia is inversely proportional to mortality. This index had nothing to do with gestational age and day age. It decreased significantly in neonatal septicemia and increased gradually after 4-12 hours of antibiotic treatment. The Inhibitory protein in the septicemia group with positive blood culture was significantly lower than that in the septicemia group with negative blood culture. When the critical value was 177n shadow L, the sensitivity, specificity, positive predictive value and negative predictive value were 89.5%, 99%, 95% and 98%, respectively.^32^

### 5. OTHER INDICATORS

Other indicators related to early diagnosis of septicemia include soluble intercellular adhesion molecule 1 (SICAM 1), universal primer 16 srRNA gene PCR, number of nucleated red blood cells and so on. LCAM 1 exists in vascular endothelial cells, fibroblasts and some epithelial cells, mediating leukocyte adhesion to endothelial cells. SlCAM 1 mainly comes from the exfoliation of adhesion molecules on the cell surface. Cytotoxins and cytokines can up-regulate the expression of SICAM 1 during infection and play an important role in the regulation of leukocyte activity in the early stage of inflammation. In the past, it has always been believed that the increase of nucleated erythrocytes is related to the increase of erythropoietin (EPO) induced by hypoxia, but Dulay AT, et al.^33^ found that the increase in the number of nucleated red blood cells in the early postnatal period is a reflection of direct exposure to inflammatory mediators in the uterus, and its release is closely related to intrauterine inflammation and cord blood IL levels, but has nothing to do with pH, EPO and cortisol levels, and has diagnostic significance for EOS. As a new diagnostic index of failed, universal primer 16 srRNA PCR has gradually attracted the attention of scholars at home and abroad. Indian scholars have confirmed that its sensitivity and specificity are more than 96% compared with the results of blood culture before antibiotic treatment, which can provide a sensitive basis for early etiological diagnosis of septicemia.^34^

Although the above indexes are related to septicemia, most of them are in the research stage. At present, the main indicators for the diagnosis of neonatal septicemia are blood WBC count and classification, and blood culture.^35^ Although many hospitals have carried out PCT detection, the clinical application is limited because of the high cost. In most cases, there is a mild association of thrombocytopenia in gram-positive organisms as compared to fungal or gram-negative organisms where there is a high incidence of thrombocytopenia.^36,37^

## METHODS OF IDENTIFYING GROUP B STREPTOCOCCI

The standard Fuller's extraction method was the most available rapid detection method. These days the suspect colonies are selected from agar plates. The carbohydrate parts of group B streptococci react with the specific part of staphylococcus and make coagulation which is macroscopically visible. The positive result shows within 6 hours. However, the majority results show positive within 24 hours.^38,39^

## TREATMENT OF NEONATAL SEPTICEMIA

### 1. ANTIBIOTIC THERAPY

Selecting appropriate antibiotics according to the results of blood culture and drug sensitivity tests is an ideal method for the treatment of neonatal septicemia, but bacterial culture cannot get results quickly. Antibiotics are often selected on the basis of experience in the clinic. It is inevitable that the pertinence is not strong.^40^

Staphylococci were the first pathogen of neonatal septicemia, of which Coagulase-negative *Staphylococcus* (CNS) was the main pathogen. However, CNS is generally resistant to penicillin, ampicillin, oxacillin and erythromycin, and has an increasing resistance rate to ceftriaxone and cefazolin. Although it is highly sensitive to vancomycin, vancomycin-resistant *Enterococci* have been detected, so ortho-cloxacillin is the first choice for CNS infection. Vancomycin should be used on the premise of an etiological basis. Methicillin-resistant CNS (MRCNS) and methicillin-resistant Staphylococcus aureus (MRSA) infections are resistant to many antibiotics, sensitive to vancomycin, and partially sensitive to amikacin, gentamicin, rifampicin, tetracycline and ofloxacin. However, due to the limitation of neonatal medication, only vancomycin can be used as the first choice for MRCNS and MRSA infection.^41^

The third-generation cephalosporins such as cefotaxime, ceftazidime and ceftriaxone can be used for Gramnegative bacilli that do not produce extended-spectrum B-lactamase (ESBLS). For those producing ESBLS, compound dosage forms with synergists, such as cefoperazone/sulbactam, or other antibiotics stable to ESBLS, such as imipenem, meropenem, etc, were used, but these drugs were expensive and were not used as first-line drugs.^42,43^

Group B *Streptococcus* and *Escherichia coli* are the most common causes of both early- and late-onset sepsis, approximately two-thirds of early-onset infections. The combination of ampicillin and gentamicin is effective in treating most common organisms. In the case of neonates with suspected meningitis the addition of expanded-spectrum cephalosporin like cefotaxime to ampicillin and gentamicin.^44^

Early-onset pneumonia before seven days of age is treated with empiric coverage with ampicillin and gentamicin. Third-generation cephalosporin should generally not be used for early-onset sepsis or pneumonia. carbapenems are not used generally due to carbapenem-resistant Enterobacteriaceae.^45^

### 2. EMOLLIENT THERAPY

One randomised clinical trial showed that the application of topical emollient reduces the chance of bloodstream infection in preterm infants. But it does not affect mortality and is insignificant in the prevalence of sepsis. It shows local irritation and is not further recommended.^46,47^

## TREATMENT PROGRESS: SOME ADJUVANT TREATMENTS ARE BEING EXPLORED

Since the 1980s, intravenous immunoglobulin (IVIG) has been widely used in the clinical prevention and treatment of septicemia in premature infants, and a large number of articles about its effectiveness have been published.^48,49^ However, the results of the study conducted by the Neonatal Collaborative Network in 1994 showed that IVIG could not reduce the incidence of nosocomial infection, and the benefits of IVIG for anti-infection were still uncertain. Since MRCNS is the main pathogen of LOS, immunoglobulin (INHA21) is extracted from donor blood with a high titer of staphylococcal antibody for the prevention and treatment of LOS. A multicenter, randomised, doubleblind, and phase III clinical observation was conducted in 1983 premature infants with BW500-1250 g in the United States. INHA21750 mg/kg or placebo were given respectively. The chance of Staphylococcus aureus septicemia and Candida infection and mortality decreased in the treatment group, but the difference was not statistically significant.^50^

Severely infected newborns quickly deplete the limited bone marrow colony-stimulating factor (BM-CSF) in the bone marrow storage pool, resulting in granulocytopenia. BM-CSF can increase the absolute value of neutrophils and enhance the chemotaxis, phagocytosis and respiratory burst response of neutrophils. Prophylactic administration of granulocyte-macrophage colony-stimulating factor (GM-CSF) can prevent infection when neutropenia occurs in newborns with gestational age less than 32 weeks (<1.7x10^9^ / L) or is at risk of postnatal neutropenia.^51^

In addition, because premature infants are often treated with antibiotics, it is easy to cause intestinal flora imbalance and increase the risk of septicemia and neonatal necrotizing enterocolitis (NEC). Preterm infants are also prone to feeding intolerance. Probiotics improve nutritional status by improving intestinal tolerance to feeding, reducing the need for parenteral nutrition, and inhibiting the growth of intestinal pathogenic bacteria. The seventy-one barrier effect of intestinal mucosa on bacteria and their products was increased, and protective immunity was up-regulated. Probiotics may reduce the incidence of septicemia, NEC and the use of antibiotics, but this conclusion is still controversial by foreign scholars.^52^

Neonatal septicemia often has coagulation dysfunction, which leads to multiple organ failures. Active protein C can inhibit thrombin production and improve microcirculation in this process. Polish scholars have successfully treated a full-term newborn with septicemia with multiple organ failure by using active protein C as adjuvant therapy. All blood coagulation indexes returned to normal after 6 hours of treatment, and the children recovered after 4 days of treatment.^53^

Although active protein C can reduce the mortality of children with severe septicemia, there are adverse reactions to bleeding. De Carolis MP, et al. reported that a premature infant with 28 days of severe septicemia achieved good results after 96 hours of inactivated protein C treatment. Therefore, it is recommended that active protein C can be used in the adjuvant treatment of severe neonatal septicemia.^54^

Although there are more and more adjuvant treatments for septicemia, it is still based on sensitive antibiotics, and the course of antibiotic treatment is generally 1014 days. For those who are suspected of septicemia but without bacterial growth in blood culture and without meningitis, the course of antibiotics can be appropriately shortened. Comparing the effects of 4-7 days courses of antibiotics on suspected neonatal septicemia with negative blood culture, it was found that there was no difference between the two methods in improving symptoms.^55^
